# Strengthening the Surveillance and Response to Public Health Events With a One Health Approach: A Perspective From 12 Countries in Latin America and the Caribbean

**DOI:** 10.1093/infdis/jiae629

**Published:** 2025-03-10

**Authors:** Ángel Rodríguez, Paula Couto, Alejandra Acevedo, Belsy Acosta Herrera, Olaya Astudillo, Martín Avaro, Gisela Barrera Badillo, Alfredo Bruno, Patricia Bustos, Mauricio Cerpa, Hector Chiparelli, Dulce Duron, Rodrigo Fasce, Axel Galo, Elías Guilarte, Irma López Martínez, Mariela G Martínez, Jairo Méndez-Rico, María Fernanda Olivares Barraza, Elena Penayo, María Paz Rojas Mena, Paula Rodríguez Ferrari, Marc Rondy, Viviana Sotomayor Proschle, Priscila S Born, Lidia Redondo-Bravo, Juliana Almeida Leite, Natalia Vergara Mallegas, Marta von Horoch, Andrea Patricia Villalobos Rodríguez, Andrea S Vicari

**Affiliations:** Health Emergencies Department, Pan American Health Organization, Washington, DC; Health Emergencies Department, Pan American Health Organization, Washington, DC; Chile National Influenza Center, Departamento de Laboratorio Biomédico Instituto de Salud Pública de Chile, Santiago, Chile; Cuba National Influenza Center, Departamento de Virología, Laboratorio de Influenza Instituto de Medicina Tropical “Pedro Kourí,” Ciudad de la Habana, Cuba; Health Emergencies Department, Pan American Health Organization, Washington, DC; Argentina National Influenza Center, Instituto Nacional de Enfermedades Infecciosas, Buenos Aires, Argentina; Centro Nacional de Influenza de México, Instituto de Diagnóstico y Referencia Epidemiológicos Dr Manuel Martínez Baéz, Ministry of Health, Mexico City, Mexico; Centro Nacional de Influenza de Ecuador, Instituto Nacional de Investigación en Salud Publica, Guayaquil, Ecuador; Universidad Agraria del Ecuador, Guayaquil, Ecuador; Chile National Influenza Center, Departamento de Laboratorio Biomédico Instituto de Salud Pública de Chile, Santiago, Chile; Health Emergencies Department, Pan American Health Organization, Washington, DC; Uruguay National Influenza Center, Departamento de Laboratorios de Salud Pública, Unidad de Virología, Montevideo, Uruguay; Honduras National Influenza Center, Laboratorio Nacional de Vigilancia de la Salud–Sección de Virología, Secretaría de Salud; Chile National Influenza Center, Departamento de Laboratorio Biomédico Instituto de Salud Pública de Chile, Santiago, Chile; Ministry of Health of Honduras, Tegucigalpa, Honduras; Cuba National Influenza Center, Departamento de Virología, Laboratorio de Influenza Instituto de Medicina Tropical “Pedro Kourí,” Ciudad de la Habana, Cuba; Centro Nacional de Influenza de México, Instituto de Diagnóstico y Referencia Epidemiológicos Dr Manuel Martínez Baéz, Ministry of Health, Mexico City, Mexico; Health Emergencies Department, Pan American Health Organization, Washington, DC; Health Emergencies Department, Pan American Health Organization, Washington, DC; Department of Epidemiology, Ministry of Health of Chile, Santiago, Chile; Ministry of Health of Paraguay, Asunción, Paraguay; Ministry of Health of Argentina, Buenos Aires, Argentina; Department of Epidemiology, Ministry of Health of Chile, Santiago, Chile; Health Emergencies Department, Pan American Health Organization, Washington, DC; Department of Epidemiology, Ministry of Health of Chile, Santiago, Chile; Health Emergencies Department, Pan American Health Organization, Washington, DC; Health Emergencies Department, Pan American Health Organization, Washington, DC; Health Emergencies Department, Pan American Health Organization, Washington, DC; Department of Epidemiology, Ministry of Health of Chile, Santiago, Chile; Ministry of Health of Paraguay, Asunción, Paraguay; Health Emergencies Department, Pan American Health Organization, Washington, DC; Health Emergencies Department, Pan American Health Organization, Washington, DC

**Keywords:** One Health, pandemic, respiratory virus, surveillance, SARInet

## Abstract

The Surveillance Network for Influenza and Other Respiratory Viruses (*SARInet*) has played a crucial role in improving the capacity of Latin American countries to monitor and respond to events with potential public health impact. This perspective compiles and analyzes case studies from several countries in the Americas to illustrate the challenges faced prior to the creation of *SARInet*, the interventions implemented, and the impacts achieved.

**Methodology:**

This study is based on a series of cases from 12 countries in Latin America and the Caribbean. The data have been compiled from reports, ad-hoc assessments and technical group analyses conducted before and after the implementation of *SARInet* in 2014.

**Results:**

Over the past decade, *SARInet* has been instrumental in strengthening the capacity for early detection and response to public health events in several Latin American countries. Technical cooperation in epidemiological and laboratory surveillance, personnel training, intersectoral coordination and the adoption of advanced technologies have improved the countries' capacity for timely detection and management of epidemic and pandemic outbreaks.

**Conclusions:**

The experience of the countries shows that regional collaboration and the strengthening of local capacities are essential to face future public health challenges.

Since 2020, the region of the Americas has recorded several outbreaks of avian influenza A(H5N1) [1]. These outbreaks, which have involved infections in different animal species, including humans, have highlighted the need to develop and maintain robust, integrated surveillance systems and rapid response capabilities to address zoonotic threats with pandemic potential [1, 2].

In this perspective, we discuss the advances in the Americas in preparation, early detection, and response capacities by looking at the lessons learned from past public health emergencies of international concern (PHEICs), such as the A(H1N1) 2009 and COVID-19 pandemics. We conclude that a One Health approach—recognizing the interconnectedness of human, animal, and environmental health—is essential for effective early detection and control of emerging infectious diseases.

## SURVEILLANCE IN THE AMERICAS: A DECADE OF PROGRESS

Surveillance progress in the countries of the Americas has been important and has been achieved mainly by indicator-based surveillance through a network of sentinel sites for syndromic surveillance of influenza-like illness and severe acute respiratory infections (SARIs), National Influenza Centers, and reference laboratories [3]. It is therefore necessary to strengthen complementary surveillance systems, such as event-based surveillance, to expand national capacities for early detection, rapid risk assessment, effective information sharing, integration with surveillance of zoonotic influenza cases, and implementation of effective prevention and control measures.

The Severe Acute Respiratory Infections Surveillance Network (SARInet) was established in 2014 by the Pan American Health Organization (PAHO) with the objective of strengthening sentinel surveillance of SARIs and improving the capacity for early detection and response to potential outbreaks and pandemics in the Americas. Over the past 10 years, SARInet has provided a platform for regional cooperation among ministries of health, reference laboratories, sentinel hospitals, and partner institutions. It promotes exchange of knowledge, experiences, and best practices and facilitates capacity building through training, technical assistance, and provision of resources and inputs [3].

To learn more about the added value of regional cooperation facilitated by SARInet in the past decade, we contacted all SARInet member countries and asked them to provide case studies describing the impact of the network in strengthening surveillance and response capacity to epidemic and pandemic events. PAHO provided the countries with instructions and guiding questions for the development of the case studies, and answers were sent as narrative responses in free text (supplementary materials). Of 33 SARInet member countries contacted, we received individual case studies from 12 countries: Argentina, Chile, Costa Rica, Cuba, Dominican Republic, Ecuador, Panama, Paraguay, Peru, Colombia, Mexico, and Uruguay. We reviewed and synthesized all answers into overarching themes, which were then integrated into this Perspectives piece.

## LEARNING FROM PAST PHEICS

Two pandemics recorded in the last 20 years, the influenza A(H1N1) 2009 pandemic and the COVID-19 pandemic, have had a great impact on regional public health, health care systems, and society, showing strengths and shortcomings in current response frameworks [3–5].

More precisely, the influenza A(H1N1) 2009 pandemic highlighted the importance of assessing severity at early stages and ensuring rapid vaccine development and distribution. In terms of shortcomings, it exposed gaps in response coordination and the need for better integration of surveillance data from different components, such as virologic, clinical-epidemiologic, vaccination, and health services. To bridge these gaps, networks such as SARInet were established with the aim to implement and strengthen the surveillance in ambulatory and severe cases through hospital-based settings and to provide a platform for regional cooperation and capacity building for preparedness and response.

The COVID-19 pandemic further stressed the need for early diagnosis to provide prompt treatment and stop transmission, genomic surveillance to detect and monitor new variants, and real-time data sharing to assess risk and implement control measures. According to member states who responded to the consultation, countries that had robust sentinel surveillance systems in place, as supported by networks such as SARInet, were well equipped to identify and respond to the emergence of SARS-CoV-2 variants.

The recent threats of events at the human-animal interphase, including zoonotic influenza, force us to reflect on the current capabilities at the regional level and the need to strengthen the preparation based on lessons learned from previous public health emergencies. A key lesson to be derived from past experiences is the importance of coordinating the response through an incident manager command system to save lives, stop transmission, and ensure the continuity of essential health services in times of crisis. Studies have shown that PHEICs can significantly affect routine health services, resulting in decreased health care utilization and challenges in managing other communicable diseases [6].

The case studies provided by the countries showed that a network such as SARInet can serve as a basis for strengthening the early detection and strengthening of genomic epidemiology, and the Regional Workshop on Lessons Learned During the COVID-19 Pandemic [7] conducted by SARInet concluded that preparedness and response require interprogrammatic, multisectoral work. Allocation of resources to the budgets of other sectors involved in health emergencies, on the basis of well-developed preparedness plans, facilitates timely access to medicines, vaccines, and supplies during public health emergencies. Through various strategies and lines of action, the network has helped countries overcome preexisting challenges and deficiencies and establish more robust, sensitive, and timely epidemiologic and laboratory surveillance systems (Table 1). Finally, the challenges faced during past and current emergencies have also shown the critical importance of early detection to enable timely response for containment purposes.

**Table 1.** Public Health Impact of SARInet Technical Collaboration

Category	Details	Countries for Which the Theme Was Mentioned
Surveillance and monitoring	Many countries have established or enhanced sentinel surveillance systems for respiratory viruses. These systems have been integrated with broader public health frameworks, enabling early detection of outbreaks such as H5N1 avian influenza and COVID-19, thus improving public health response.	Argentina, Chile, Cuba, Ecuador, Paraguay, Panama, Uruguay
Laboratory strengthening	Across the region, a key outcome was the enhancement of laboratory capacities. Countries have upgraded their laboratory capacities by incorporating molecular diagnostic tools (eg, real-time reverse transcription–polymerase chain reaction), decentralizing molecular diagnostics, and integrating genomic sequencing. Other elements, such as provision of supplies and reagents, training of staff, and development of diagnostic protocols, have been instrumental for the detection and surveillance of respiratory viruses. The exchange of avian influenza A/H5N1 samples from animals and humans with the WHO Collaborating Centers has been reinforced, thereby facilitating the risk assessments and decisions for the selection of vaccines and strengthening pandemic preparedness.	Chile, Cuba, Uruguay, Paraguay
Capacity building	Capacity-building efforts have included training in epidemiology, molecular techniques, and bioinformatics, as well as workshops of lessons learned and intersectoral response across the region. These initiatives have ensured the readiness of health personnel to respond to epidemics and pandemics, keeping an emphasis on intersectoral cooperation using a One Health approach.	Ecuador, Panama, Paraguay, Uruguay
Impact and outcomes	The collective efforts in the region have resulted in improved public health outcomes, including the response to the COVID-19 pandemic and avian influenza outbreaks in Latin America and the Caribbean. All together, these initiatives have demonstrated the value of intersectoral approaches to public health, with enhanced capacity to respond to emerging health threats.	Argentina, Chile, Ecuador, Panama

Abbreviation: SARInet, Severe Acute Respiratory Infections Surveillance Network.

## IMPORTANCE OF STRENGTHENING EARLY DETECTION

The case studies revealed a substantial and multifaceted impact of SARInet in strengthening the capacity of early detection, surveillance, and response to public health events in the region. Several common themes and achievements were identified.

### Development of Early Warning Systems

The implementation and strengthening of sentinel surveillance of SARI with the support of SARInet have allowed countries to have robust systems for trend monitoring of respiratory virus activity.

Countries covering all subregions of the Americas highlighted how this sentinel surveillance enabled them to detect SARS-CoV-2 cases early and monitor the COVID-19 pandemic. Tools implemented by SARInet, such as the pandemic influenza severity assessment (PISA), have been key to assessing the risk of events. In the region of the Americas, only 8 countries report routine PISA indicators: Bolivia, Canada, Colombia, Guatemala, Mexico, United States, Paraguay, and Suriname. The implementation of PISA in the region was carried out through regional, subregional, and country-level training, using as a basis the World Health Organization (WHO) guideline, which includes the methodology for the selection and development of the thresholds. Internally, countries use the indicators as part of the weekly assessment of the season of greatest activity for respiratory viruses, and some countries, such as Chile and Paraguay, report it within their weekly bulletin. PAHO updates the PISA parameters weekly for all the countries that report the relevant information via FluNet and FluID; these are published weekly in PAHO's regional influenza bulletin and are used in internal risk assessment discussions.

Strong indicator-based systems lay the groundwork for increased surveillance capabilities beyond the sentinel strategy, with the objective of early detection of outbreaks and the detection of unusual changes in respiratory viral diseases. This was particularly evident during the acute phase of the COVID-19 pandemic, where countries such as Chile, Costa Rica [8], Dominican Republic, Ecuador, Paraguay, Mexico, and Uruguay adapted the structure, procedures, and online platform of their system for sentinel surveillance of viral respiratory diseases to expand nationwide and conduct surveillance of COVID-19. Notably, countries carried out the scaling and de-escalation of indicator surveillance differently, which evidenced the need for complementary surveillance approaches that are sustainable and representative [7]. This finding highlighted the lack of representativeness of outpatient surveillance to assess transmissibility, as well as the absence of severity data, such as hospital capacity, which some countries (eg, Uruguay) were tracking before the COVID-19 pandemic but most later used as an indicator during the pandemic. SARInet, through PAHO/WHO, is collaborating with countries to strengthen and complement surveillance systems using the WHO Mosaic initiative [9].

### Laboratory Capacity Building

SARInet has substantially improved the capacity of national influenza laboratories and public health laboratories for the diagnosis and surveillance of respiratory viruses. This has been achieved through the provision of equipment, reagents (through the International Reagent Resource) and supplies, technology transfer (eg, real-time reverse transcription–polymerase chain reaction, sequencing), development of new diagnostic protocols and algorithms, and ongoing staff training. Countries in the subregions of Central America, Caribbean, and South America highlighted the importance of this support for the timely detection of respiratory viruses, including the rapid implementation of tests for SARS-CoV-2, the provision of reagents to national laboratories during the COVID-19 pandemic via the International Reagent Resource, and the establishment of subsequent genomic surveillance. To date, SARS-CoV-2 genomic data have been uploaded to GISAID by 53 countries and territories across the Americas.

### Integration of Intersectoral Surveillance

SARInet promotes and facilitates technical intersectoral collaboration and resources, particularly between the human and animal health sectors, following the One Health approach and with partners such as WHO, Food and Agriculture Organization, World Organization for Animal Health, WHO Collaborating Centers, US Centers for Disease Control and Prevention, and St Jude Research Hospital [10]. This collaboration has enabled better detection, investigation, and response to zoonotic events, and it has contributed to the development of national plans to respond to public health threats such as avian influenza A(H5N1). For example, Chile and Ecuador, the only 2 countries in Latin America that recorded avian influenza A(H5N1) infections in humans through SARI surveillance [11, 12], identified the value of regional and global guidelines [13–15], trainings, and webinars for integration and response to the event in an intersectoral manner. They also highlighted the collaboration of the SARInet network for the submission of samples to the WHO Collaborating Centers for further characterization of cases.

### Generation of Evidence for Policy and Control Measures

Surveillance data strengthened through SARInet have helped inform decision making and guide implementation of prevention and control measures in countries of the Americas. For example, burden of disease and vaccine effectiveness studies [16, 17] conducted in the region have guided vaccination strategies [18]. The COVID-19 pandemic also showed that epidemiologic and virologic information guide public health policy and the implementation of public health measures to strengthen the response.

### Value of Regional Cooperation

According to these case studies, SARInet has strengthened national capacities and bilateral collaboration among countries in the past 10 years. Countries that were consulted recognized the added value of SARInet as a platform for regional cooperation. The exchange of experiences, knowledge, and best practices through regional meetings [19], intersectoral consultations [20], and ad hoc meetings for outbreak response [21] was highly valued, as were the direct ongoing technical assistance, thorough SARInet technical groups, and provision of standardized and harmonized protocols.

## EMBRACING A ONE HEALTH APPROACH

Beyond detection, the data generated through strengthened surveillance have been crucial in guiding evidence-based prevention and control policies and interventions. Burden-of-disease studies, evaluations of vaccine effectiveness, analyses of risk factors, and epidemiologic characterization of events have provided strategic information for informed decision making in some countries. While these have been great advances in the region, there is still a need to continue integrating surveillance with clinical data to assess severity and by animal and environmental surveillance components.

The promotion of intersectoral collaboration, particularly within the One Health approach, was another important aspect highlighted in the consultation. Given that many emerging respiratory disease threats are of zoonotic origin, such as avian influenza, coordination and cooperation mechanisms among the human, animal, and environmental health sectors are crucial [22]. SARInet started fostering this integration in 2017 with the first intersectoral meeting at the regional level conducted in Washington, DC. This meeting led to the development of action plans to strengthen surveillance and information exchange and to the creation of a road map for the implementation of rapid response workshops for zoonotic events, which was carried out at the regional level at the Fifth SARInet Meeting and later in countries such as Bolivia, Suriname, and the Dominican Republic. Also, intersectoral lessons learned were discussed and shared during the 2024 SARInet Plus regional meeting in Mexico [23]

The recent call to establish an Intersectoral Commission for the Prevention and Control of Zoonotic Influenza in the Americas [24] represents a promising step in this direction. This commission, to be led by PAHO, aims to bring together diverse stakeholders to strengthen preparedness and response capabilities across the region.

Key priorities for this commission should include the following:

- Mapping and enhancing national and regional surveillance capacities across human, animal, and environmental sectors
- Establishing mechanisms for real-time data sharing and integrated analysis
- Conducting regular joint risk assessments to identify and prioritize emerging threats
- Developing and testing cross-sectoral preparedness and response plans
- Strengthening laboratory networks and genomic surveillance capabilities
- Addressing gaps in coordination and response through multidisciplinary simulation exercises
- Serving as a platform for regional and global stakeholders to collaborate on surveillance, research, and response strategies
- Securing high-level political commitment and adequate resources for One Health initiatives

These technical priorities were addressed on 30 October 2024 during the launch of the intersectoral working groups for surveillance and risk assessment of zoonotic influenza in the Americas [25].

## LIMITATIONS

This perspective presents some limitations. First, it is important to note that only 12 of the 33 SARInet member countries contacted in the region provided case studies, which limits the representativeness of the findings and affects the comprehensive understanding of the regional capacity and added value of the network. Consequently, the results may not accurately reflect the diversity of challenges and capacities across the Americas.

Second, critical factors linked to infectious disease dynamics, such as the impact of climate change, migration, and urbanization and the increased mobility of people, were not addressed, since SARInet collaboration focused on epidemiologic and laboratory surveillance in the past years. While SARInet has made significant contributions to capacity building in surveillance and laboratory, intersectoral collaboration has yet to incorporate an integrated framework to address emerging threats posed by environmental and social determinants of health. This gap reveals the need for a more comprehensive strategy for the upcoming years in the region.

## CONCLUSION

Strengthening regional capacity to detect, assess, and respond early to zoonotic influenza threats will require sustained commitment and investment from countries across the Americas. By embracing a One Health approach and learning from past experiences, we can enhance our preparedness for future pandemics and zoonotic disease outbreaks. The proposed influenza commission represents a significant step toward achieving these goals, ensuring a coordinated and effective response to influenza and other zoonotic threats.

## Supplementary Data

Supplementary materials are available at *The Journal of Infectious Diseases* online (http://jid.oxfordjournals.org/). Supplementary materials consist of data provided by the author that are published to benefit the reader. The posted materials are not copyedited. The contents of all supplementary data are the sole responsibility of the authors. Questions or messages regarding errors should be addressed to the author.

## Supplementary Material

jiae629_Supplementary_Data

## Notes

***Acknowledgments.*** We thank the member states of PAHO for contributing to the regional and global respiratory virus surveillance through the Global Influenza Surveillance and Response System/WHO and for all the collaboration and resilience of the epidemiologic teams and laboratory teams, including the National Influenza Center and National Public Health Laboratories as part of SARInet Plus. We give special thanks to those in charge of surveillance of influenza and other respiratory viruses in the ministries of health and National Influenza Centers of Colombia, Costa Rica, Dominican Republic, Ecuador, Mexico, and Panama for having contributed with country case studies for the development of this article.

We are also grateful for the support of personnel at WHO headquarters, the PAHO/WHO Regional Office, the Pan American Center for Foot-and-Mouth Disease and Veterinary Public Health, and PAHO/WHO Country Offices for the technical cooperation provided in the region to respiratory viruses, seasonal epidemics, and pandemics. Also, we give special thanks to the WHO Collaborating Center at the US Centers for Disease Control and Prevention for all technical collaboration and support to the establishment of the SARInet network and support through the International Reagent Resource, assisting the laboratories with reagents needed and updated primers and probes for influenza for the National Influenza Centers and reference laboratories.

***Author contributions.*** Manuscript conception and design: A. R. Data collection: A. A., A. B. C., A. G., B. A. H., D. C., E. P., E. G., G. B. B., H. C., I. L. M., M. F. O. B., M. P. R. M., M. v. H., M. A., N. V. M., P. B., P. R. F., R. F., and V. S. P. Analysis and interpretation of results: A. R. Draft manuscript preparation: A. R., O. A. All authors critically reviewed the results and draft and final versions of the manuscript for intellectual content.

***Supplement sponsorship.*** This article appears as part of the supplement “Advancing Respiratory Virus Surveillance and Preparedness in the Americas: A Decade of SARInet's Impact,” sponsored by the Pan American Health Organization.

***Financial support.*** This work was supported by the United States Centers for Disease Control and Prevention through a cooperative agreement with the Pan American Health Organization/World Health Organization.

***Potential conflicts of interest.*** All authors: No reported conflicts. All authors have submitted the ICMJE Form for Disclosure of Potential Conflicts of Interest.
