# Supplementary material for: Strengthening the Surveillance and Response to Public Health Events With a One Health Approach: A Perspective From 12 Countries in Latin America and the Caribbean
Source: J Infect Dis. 2025 Mar 10;231(Suppl 2):S108–13. doi: 10.1093/infdis/jiae629 (PMC11892000; doi:10.1093/infdis/jiae629)
Supplement: jiae629_Supplementary_Data [file jiae629_supplementary_data.pdf]

Guía para presentar un estudio de caso

Fortalecimiento de innovaciones para la detección temprana y respuesta a eventos de salud pública (incluyendo enfermedades respiratorias inusuales) a través de SARInet en los últimos diez años

Instrucciones: Por favor, siga las siguientes pautas para presentar un estudio de caso concreto que ejemplifique cómo SARInet ha fortalecido la detección temprana y respuesta a eventos epidémicos y pandémicos en su país. Solicitamos este documento no exceda más de 2 páginas, y se presente de la forma más clara y concisa posible. El equipo regional se pondrá en contacto con usted(es) para solicitar más información conforme se avance el proceso de escritura del artículo científico.

Contexto:

- Describa brevemente la situación epidemiológica previa al establecimiento de SARInet en su país.
- ¿Qué desafíos o deficiencias se enfrentaban en la detección y respuesta a eventos epidémicos y pandémicos antes de SARInet?

Resultados e Impacto:

- Identifique y analice tres puntos principales que ilustren el impacto de SARInet en el fortalecimiento de la detección temprana y respuesta a eventos epidémicos y pandémicos en el país.
- **Para cada punto, responda a las siguientes preguntas:**
    - **Qué:** ¿Qué acción específica se llevó a cabo o se facilitó mediante SARInet? ¿Qué tecnologías, capacidades o infraestructuras se han fortalecido a través de SARInet?
    - **Cómo:** ¿Cómo contribuyó SARInet a esta acción o facilitó su implementación?
    - **Cuándo, Dónde:** ¿En qué momento y lugar se produjo esta acción y el resultado o impacto?
    - **Cuál fue el impacto/resultado:** ¿Qué beneficio o fortalecimiento trajo consigo esta acción en términos de detección temprana y respuesta a eventos epidémicos y pandémicos? ¿Qué ha aprendido el país a través de esta experiencia?

Referencias y recursos adicionales *(si aplica)*
